# Methyl jasmonate and crude extracts of *Fusarium solani* elicit agarwood compounds in shoot culture of *Aquilaria malaccensis* Lamk.

**DOI:** 10.1016/j.heliyon.2021.e06725

**Published:** 2021-04-16

**Authors:** Ahmad Faizal, Rizkita Rachmi Esyanti, Nisaa Adn'ain, Silmi Rahmani, Alda Wydia Prihartini Azar, Iriawati, Maman Turjaman

**Affiliations:** a Plant Science and Biotechnology Research Group, School of Life Sciences and Technology, Institut Teknologi Bandung, Jalan Ganeca 10, Bandung 40132, Indonesia; b Forest Microbiology Research Group, R & D Centre for Forest, Environment & Forestry Research, Development, and Innovation Agency (FORDA), Ministry of Environment and Forestry, Jalan Gunung Batu 5, Bogor, 16680, Indonesia

**Keywords:** Agarwood, *Aquilaria malaccensis*, Elicitation, *Fusarium solani*, Methyl jasmonate, Shoot culture

## Abstract

Agarwood forms in the heartwood of trees in the family Thymelaeaceae in response to wounding, infection, or other stresses. Its formation is random and takes decades in natural populations, which are harvested for their aromatic compounds. This harvest has led to declining population, and many agarwood producing trees are considered endangered. Therefore, an alternative source would be desirable. We established an in vitro shoot culture method for one agarwood species, *Aquillaria malaccensis*. Agarwood production was elicited by introducing methyl jasmonate (MeJA) and crude extracts of *Fusarium solani* into the liquid culture medium. A high concentration of MeJA resulted in necrotic shoot tissue, while application of the crude extracts had no effect on growth of the shoots. Interestingly, gas chromatography-mass spectrometry (GC-MS) analysis of MeJA-treated shoots revealed the presence of several agarwood compounds, including sesquiterpenes and chromone derivative. In addition, GC-MS analysis of shoot-treated with the extracts revealed the presence of alkanes, aromatic compounds, and fatty acid derivatives. It may be that different elicitors induce the production of different compounds in *A. malaccensis* in vitro shoot cultures and could be used to manipulate the accumulation of different products in culture.

## 1 Introduction

Agarwood is a fragrant, resinous wood that forms in the heartwood of tress in the family Thymelaeaceae as a response to physical wounding, infection, or other stresses (Tan et al., 2019). The term also applies to resin produced in the wood. The resin is only produced when the tissues of the tree are damaged. The physical damage caused by boring insects will be followed by microbial infection, including fungi. Fungi is believed to be the main microorganism which could induce plants defense mechanisms by entering through the wound. Agarwood compounds will be produced as a result of defense mechanism to the fungi as pathogens (Rasool and Mohamed, 2016).

Agarwood is one of the most important non-timber forest products in the international market. High-quality agarwood may cost as much as US$ 100,000/kg (Naef, 2011). The agarwood is a component of incense, which is used in religious practices and it is used in holistic theraphies. It is also a component of some pharmaceuticals and cosmetics, therefore important for these industries (Naziz et al., 2019; Nasution et al., 2019; Liao et al., 2018; Kalra and Kaushik, 2017). Agarwood trees are native to India, South Asia, and Southeast Asia, and many are in one of two genera: *Aquilaria* and *Gyrinops*. Herein, we focus on the genus *Aquilaria.*

In their natural habitat, *Aquilaria* trees require a considerably long time to form agarwood. The high demand for agarwood coupled with this long production time has led to overharvest and depletion of this natural resource. Particularly, this threat was recognized for *Aquillaria malaccensis*, and it has been listed in Appendix II (species that are not necessarily now threatened with extinction, but that may become so unless trade is closely controlled) by the Convention of International Trade in Endangered Species of Wild Fauna and Flora (http://checklist.cites.org). This species is also categorized as vulnerable by the International Union for Conservation of Nature (IUCN) red list and included in The World List of Threatened Trees (http://www.iucnredlist.org).

*A. malaccensis* is the principal producer of agarwood as it gives superior quality due to its chemical constituents and high yield. This species is an agarwood-producing tree which commonly used for perfumery, medicine, and religious traditions in Asia and Middle East (Chhipa et al., 2017; Liu et al., 2017; Lee and Mohamed, 2016). Clearly, large-scale harvesting of the natural population of *A. malaccensis* can not continue at present, and an alternative source of agarwood is needed. In vitro culture of cells, tissues, and organs, is commonly used for rapid propagation of plants and could perhaps, meet this need. In culture, resin production would be elicited by inoculating the cultures tissue by biotic means or chemical elicitors, and it might even be possible to manipulate the production of several secondary metabolites. Several researcher have examined in vitro elicitation of agarwood compounds in cell suspension cultures. Methyl jasmonate (MeJA) increased the production of agarwood compounds in *Aquilaria* cultures (Okudera and Ito, 2009). MeJa is a derived of jasmonate, a class of endogenous plant growth regulator. Numerous plants responses to synthesis and presence of MeJA. When the plants were wounded, MeJA would be secreted as a gas and could activate plant defense mechanism (Rohwer and Erwin, 2008).

Furthermore, biotic elicitors, specifically crude extracts of *Trichoderma* (Jayaraman and Mohamed, 2015) or *Fusarium* (Sen et al., 2017), increased the production of agarwood constituents in *A. malaccensis* cell suspension cultures. *Fusarium* spp. are endophytic fungi which known to have an important role in agarwood formation (Faizal et al., 2020; Subasinghe et al., 2019; Tian et al., 2013; Mohamed et al., 2010). To our knowledge, though, no studies of agarwood production in *A. malaccensis* shoot cultures have been performed. Thus, we evaluated the effect of MeJA and *Fusarium solani* crude extract in shoot cultures of *A. malaccensis*. *F. solani* was opted because this fungi is a common biotic elicitor of agarwood in natural population of *Aquilaria* and has been used for the production of artifical agarwood in *A. malaccensis* (Faizal et al., 2017).

## 2 Materials and methods

### 2.1 Plant material

Shoots of *A*. *malaccensis* were acquired through axillary buds multiplication according to a method developed by Esyanti et al. (2019). Axillary shoots were incised and subcultured on MS solid medium (Murashige and Skoog, 1962) supplemented with 0.5 ppm benzyladenine (BA). Shoot cultures were incubated for 3 weeks in a growth camber at room temperature under light intensity of 700 lux for 12 h followed by 12 h dark. Elongated shoots were subsequently subcultured on MS medium without hormone. The shoot was subcultured every month for as long as 5 months. The subcultured shoot was then acclimatized on half-strength MS liquid medium for a week.

### 2.2 Preparation of fungal crude extract

Two strains of *F. solani* from FORDA (Santoso et al., 2011) isolated from Gorontalo (strain GSL1) and Jambi (strain GSL2) provinces were inoculated on potato dextrose agar (PDA) and incubated at 28 °C for 3 d. Approximately 1 cm^2^ of mycelial plugs were cut and transferred into 100 mL potato dextrose broth and incubated on a rotary shaker (100 rpm) at 28 °C for 3 weeks based on trial test of inoculant production from FORDA (Santoso et al., 2011). Fully grown mycelia were harvested on Whatman filter paper No. 1 and washed twice with sterile distilled water. Mycelia were subsequently freeze dried, grinded with liquid nitrogen, and autoclaved at 121 °C for 20 min before use (Jayaraman and Mohamed, 2015).

### 2.3 Treatment to elicit agarwood formation

Shoots of *A. malaccensis* were grown in thin layer culture using a 100 mL Erlenmeyer flask containing 10 mL of half-strength MS liquid medium. Shoot cultures were incubated on a rotary shaker at 60 rpm with the same conditions described above until harvested. After 10 days, shoots were subcultured to new MS liquid medium supplemented with 8 mg L^−1^ fungal crude extract according to Jayaraman and Mohamed (2015) or different concentration of MeJA (12.5, 25, 50, 100, and 150 μM). Untreated shoots of *A. malaccensis* served as the control group. Fungal-treated cultures were harvested 15 days after treatment. Culture treated with MeJA were harvested 1, 3, and 5 weeks after treatment.

### 2.4 Extraction and analysis of agarwood compounds in shoot extracts

Shoot of *A. malaccensis* were freeze dried and extracted using ethyl acetate solvent with ratio 1:10 (m/v). Samples were homogenized by shaking at 100 rpm for 24 h and diluted twice by adding the solvent until the ratio between sample and solvent reached 1:30 (m/v). Samples were concentrated overnight and centrifuged at 1,000 rpm for 10 min. Supernatants were collected and injected into GC-MS device (GCMS-QP2010 Ultra, Shimadzu, Europe). GC-MS analysis were done at an interface temp 250 °C, a solvent cut time of 3 min and an end time of 61 min. The identified compounds from GC-MS data were used for metabolomics data analysis using R package and displayed as a heatmap.

## 3 Results and discussion

### 3.1 The effect of MeJA on elicitating agarwood formation

**Figure 1:**
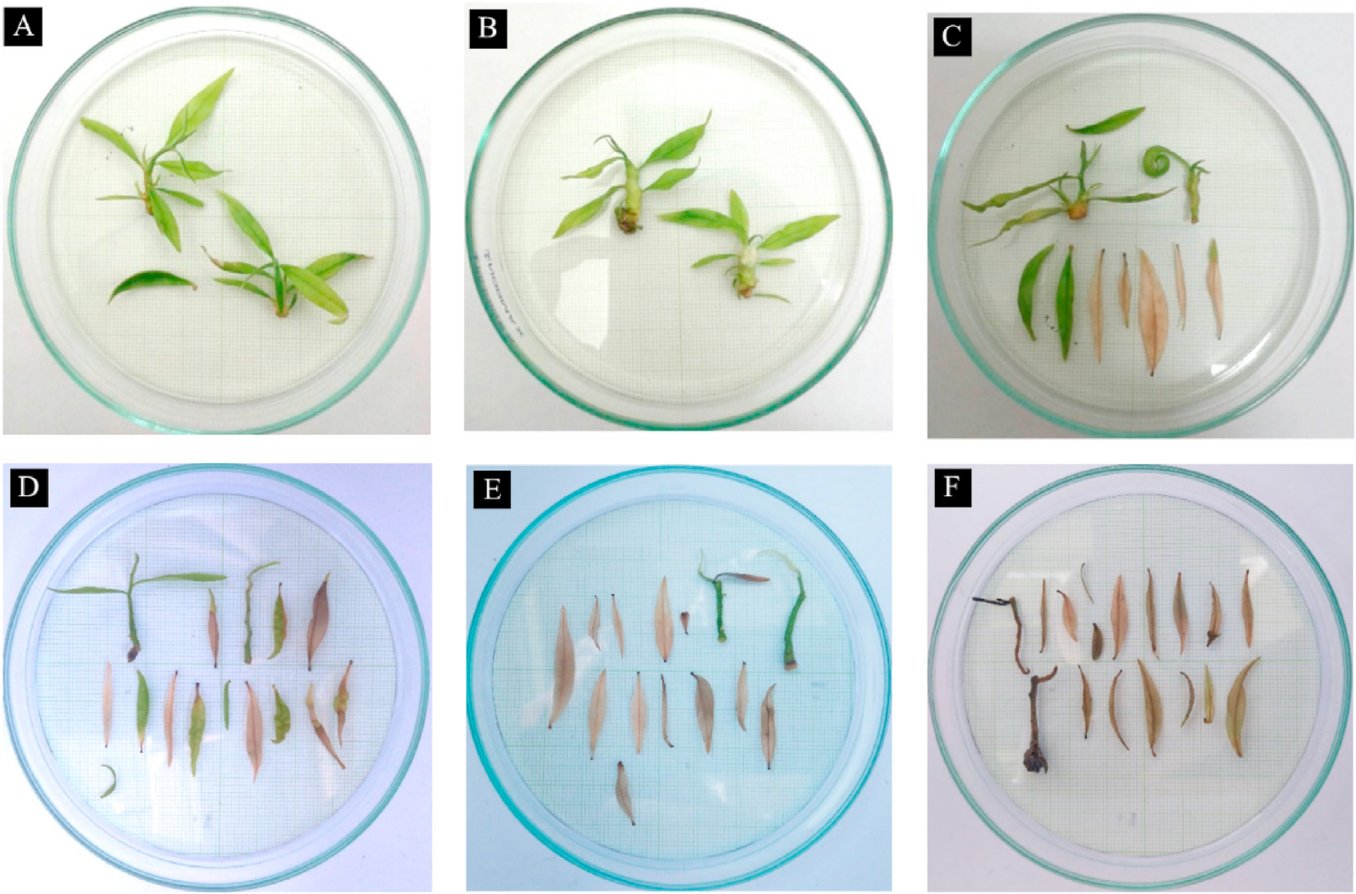
*A. malaccensis* shoots after treatment with methyl jasmonate (MeJA) for 3 weeks (A) Control, (B) 12.5 μM, (C) 25 μM, (D) 50 μM, (E) 100 μM, and (F) 150 μM.

Treatment with high concentration of MeJA affected shoot growth in vitro. At the lowest concentration of MeJA (12.5 μM), there were no visible effect on shoots during the 3 weeks of treatment, but when MeJA concentration was 50–150 μM, we observed leaf senescence and shoot necrosis (Figure 1) and the shoots did not recover when the plants were transferred into fresh MS medium without MeJA.

**Figure 2:**
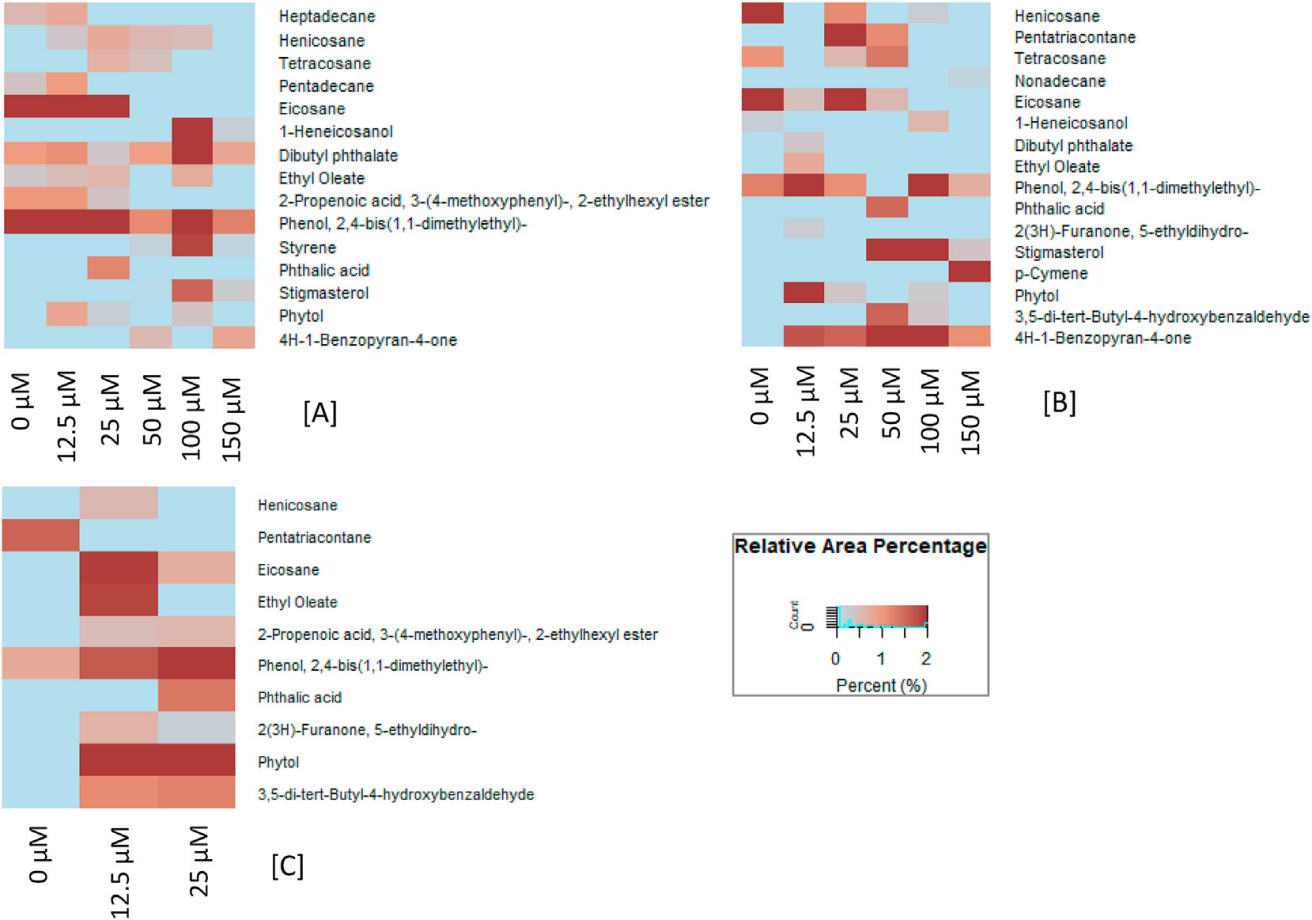
Heatmap of chemical constituents in *A. malaccensis* shoot culture elicited by MeJA treatment. (A) 1 week, (B) 3 weeks, and (C) 5 weeks of treatment.

From analysis with GC-MS, we detected several compounds associated with agarwood in the shoots, but these varied with respect to MeJA concentration and length of treatment (Figure 2). Similarly, these compounds have been also reported from the previous studies (Okudera and Ito, 2009; Xu et al., 2016). Indeed, when we assessed the effect of MeJA concentration and length of the treatment on secondary metabolites that accumulated, we observed striking differences (Figure 2). In the untreated, control shoots, neither aromatic nor terpenoid compounds as agarwood chemical constituents were detected until the end of treatment period. In the shoots treated with MeJA, most of the chemicals that accumulated were alkanes and alkenes and the amounts of these increased with concentration of MeJA.

The types of aromatic and terpenoid compounds produced by *A. malaccensis* shoots in culture in this study in response to MeJA were similar to the compounds reported by others from similar studies (Okudera and Ito, 2009). Particularly, we noted accumulation of aromatic styrene in shoots treated with 50 μM or more MeJA during the first week. Phthalic acid and 2(3H)-furanone, 5-ethyldihydro were only detected in shoots cultured with 25–50 μM MeJA. Culturing the shoots with 12.5; 25; 50; or 100 μM MeJA resulted in accumulation of 3.5-di-tert-butyl-4-hydroxybenzaldehyde, a sesquiterpene, by the end of treatment time.

Treatment with all MeJA concentrations has been noted to induce the formation of chromone 4H-1-benzopyran-4-one. The *A. malaccensis* shoots treated with 150 μM MeJA for one week had the more chromone than the other shoots. It is of interest to note that treatment with 50, 100, or 150 μM MeJA induced shoot necrosis and cell death during the first week of culture. Apparently, then, these necrotic tissues still permitted chromone biosynthesis in *A. malaccensis* shoots after one week of treatment. Chromone were also detected in shoots in 3 weeks of culture time of all samples treated with MeJA. We conclude that the accumulation of dead cells until 3 weeks of stress still allowed the formation of chromones in the treatment groups. Unfortunately, the increase of dead cells in the culture was causing the depletion of chromones. There is no chromone detected in all samples after 5 weeks of treatment. This was related to accumulation of dead cells after 3 weeks of treatment. Resinous agarwood accumulation in agarwood producing trees is one aspect of their defense mechanism. Therefore, external stimuli are necessary for the trees to synthesize agarwood and the defense-related compounds in it. Jasmonic acid and MeJA are signaling compound in plants defense pathway and consequently regulate secondary metabolism (Rohwer and Erwin, 2008; Öztürk et al., 2014). It is suspected that the signal of jasmonic acid produced by MeJA in shoot culture is important for *Aquilaria* species to induce the formation of self-defense compounds (Okudera and Ito, 2009; Xu et al., 2013, 2016). This background is the basis of our use of MeJA as an elicitor for agarwood formation here.

According to Sen et al. (2017), alkane compounds, for example pentatriacontane, and tetradecane, 2-methyl, mostly confer the chemical aroma of healthy *Aquilaria* trees. In this study, several of the aromatic compounds that we detected in shoot treated with MeJA, such as phthalic acid, and 2 (3H)-furanone, 5-ethyldihydro were also present in agarwood oil from *A. malaccensis* that had been elicited by inoculation with fungi (Chhipa and Kaushik, 2017).

The chromone derivative, 4H-1-benzopyran-4-one was detected in this study in all shoots treated with MeJA. This compound was also detected in *A. sinensis* agarwood that had been induced by *Colletetrichum gloeosporoides* and *Botryospheria* sp. (Tian et al., 2013). At present, the chromone biosynthesis pathway has not been elucidated. In this study, 4H-1-benzopyran-4-one accumulated in apparently dead cells, as has been previously observed in *Aquilaria* cell culture (Okudera and Ito, 2009).

### 3.2 The effect of crude extracts of *F. solani* in eliciting agarwood formation

**Figure 3:**
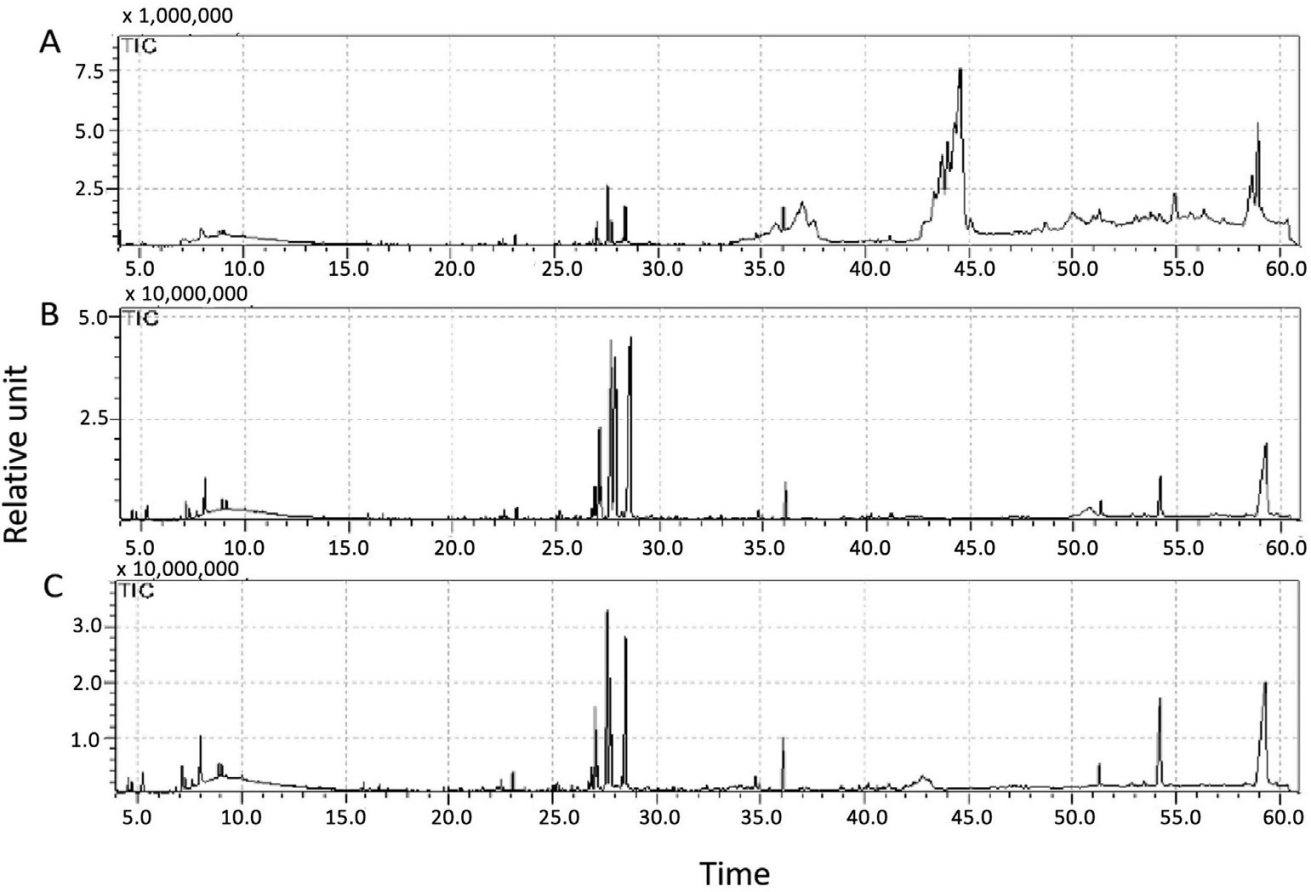
GC-MS chromatogram of control *A. malaccensis* shoots in culture. (A) untreated shoots, (B) shoots treated with *F. solani* from Gorontalo (GSL1), and (C) shoots treated with *F. solani* from Jambi (GSL2).

We analyzed the effect of *F. solani* extracs on accumulation of agarwood constitutents in cultured shoots of *A. malaccensis*. There were three treatment groups: a control group of shoots that were not treated with fungal extract, and two groups treated with *F. solani* extracts (strain GSL1 and GSL2; designated groups GSL1 and GSL2, respectively). GC-MS analysis detected a total of 12 agarwood-associated compounds in these three groups. Generally, chromatograms of shoots from the control group were different than those from groups GSL1 and GSL2, which had similar chromatograns (Figure 3). The compounds in the treated samples were, presumably, elicited by the fungal extract.

**Figure 4:**
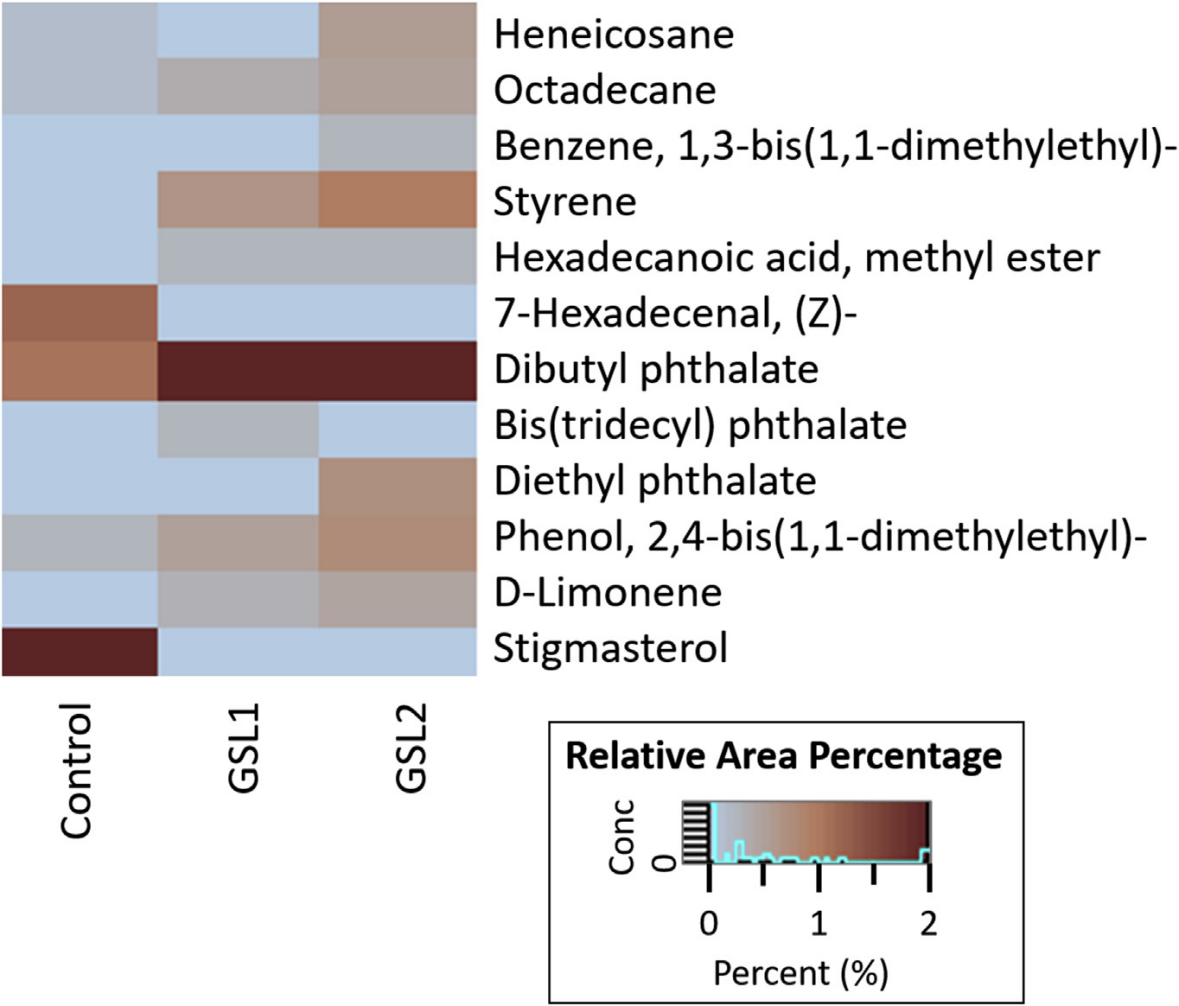
Heatmap of chemical constituents in *A. malaccensis* shoot culture elicited by *F. solani*.

Of the 12 compounds detected, many were alkanes, aromatics, or fatty acids and their derivatives. However, sesquiterpene and chromone, which are widely known as major agarwood compounds, were not present. The agarwood constituent compounds in these shoots are shown in Figure 4.

Aromatic compounds were present in all groups also, but the relative content of the fungal-treated shoots was somewhat greater than the control. The aromatic benzene, 1,3-bis(1,1-dimethylethyl)- was present only in shoots treated with strain GSL2 samples. This compound was also detected in cell suspension cultures treated with *Fusarium* crude extract by Sen et al. (2017). In a previous study, styrene was detected in *A. malaccensis* after inoculation with *F. solani* (Naef, 2011); we also detected styrene only in the fungal-treated shoots. Stigmasterol was a major compound in the untreated control shoots, but it was not present in the other groups. From the result in Figure 4, we conclude that the cultured shoots treated with fungal extracts of strain GSL2 had the greatest accumulation of garwood constituents in this study.

Shoots of *A. malaccensis* treated with crude extracts of *F. solani* accumulated fatty acids, heneicosane, and octadecane. Heneicosane has also been detected in several other agarwood-producing tissues. In *A. sinensis*, it was present both in healthy tissues and following treatment with chemical elicitors (Chen et al., 2011), and in tissues inoculated with *Lasiodiplodia* sp. or *Paraconiothyrium variabile* (Cui et al., 2013). It was also observed in cell suspension culture of *A. malaccensis* inoculated with crude extracts of *Trichoderma* or *Fusarium* sp. (Jayaraman and Mohamed, 2015; Sen et al., 2017). Octadecane was found in healthy *Aquilaria* and after inoculation with *Chaetomium globosum* or *F. oxysporum* (Tamuli et al., 2005).

Fatty acids appear to be dominant compound in extracts of both healthy and fungal-inoculated *A. agallocha* (Tamuli et al., 2005). In this study, the methyl ester of hexadecanoic acid, a fatty acid derivative, was present only in shoots treated with either strain of *F. solani*. Apparently, this compound is a common constituent of agarwood as it was detected in both artifical agarwood induced by fungal inoculation (Mohamed et al., 2014), as well as cell suspension cultures treated with *Trichoderma* extract (Jayaraman and Mohamed, 2015).

The aldehyde, 7-hexadecenal, (Z)-, was present in *Aquilaria* grown in vitro that had been treated with fungal extract (Sen et al., 2017). The acid ester, dibutyl phthalate was detected in shoots of *A. malaccensis* in our study. This compound was also observed in healthy *A. sinensis* tissues as well as naturally formed and chemically induced agarwood of this species (Chen et al., 2011), and in agarwood-producing trees inoculated by *P. variabile*, *Lasiodiplodia*, or *Xylaria* (Cui et al., 2013). Here, bis(tridecyl) phthalate was present only in shoots inoculated with the GSL1 strain of *F.* solani, while diethyl phthalate was present only in those inoculated with the GSL2 strain. Both acid esters were induced during elicitation of *A. malaccensis* cell suspension cultures with *Fusarium* extract (Sen et al., 2017).

Stigmasterol, a major component in the control shoot extracts, was also detected in healthy and wounded *A. malaccensis* (Mohamed et al., 2014). Furthermore, phenol, 2,4-bis(1,1-dimethylethyl)- was present in all shoots in our study, but its relative content was greater in the shoots treated with elicitors. This compound was present in agarwood from *A. sinensis* trees eight month after artificial induction with combination between chemically and fungal inoculation (Tian et al., 2013), as well as in cell suspension cultures of this species that had been treated with *F. solani* extracts (Sen et al., 2017). Phenolic compounds are important in the plant's self-defense system, and their levels increase in agarwood trees upon infection (Novriyanti and Santosa, 2011). This background accounts for the low phenolic content of the healthy control shoots compared to the groups inoculated with fungal extract.

D-limonene was the only terpene detected in this work. This monoterpene that has a distinct fresh citrus aroma. It was present in extracts of the shoots treated with the elicitors only, and it is reported to be only a minor agarwood component in *A. malaccensis* and *A. subintegra* (Pripdeevech et al., 2011). Marei et al. (2012) noted that limonene has potential as a natural antifungal and insecticidal agent.

The absence of sesquiterpene and chromone in the fungal-elicited shoots could be due to several possible factors, such as the fungi species used and its virulence, the form of elicitor used, and the age of culture when treated with the elicitor. The extraction method might also affect the compounds present. Most sesquiterpenes are nonpolar, volatile compounds that can diffused into culture medium or air inside the growth vessels. Finally, the is a threshold for detection and compounds may have been present at levels below this threshold.

In general, elicitation resulted in the production of more secondary metabolites than in the untreated controls. There were more of the compounds that were present in all three groups, such as octadecane and dibutyl phthalate, in the shoots treated with elicitors, and the elicitors also induced the production of several agarwood constituents that were not present in the controls. These include 1,3-bis(1,1-dimethylethyl)-benzene; styrene; methyl ester hexadecanoic acid; and D-limonene. This result may provide evidence that the elicitors can be selected to target enhanced production of particular secondary metabolites production in *A. malaccensis* shoot cultures.

## 4 Conclusion

This study contributes to the utilization of elicitors to induce agarwood compounds in cultured shoots of *A. malaccensis*. Notably, MeJA treatment induced the agarwood chemical constituents sesquiterpene and a chromone derivative, while these were not detected in shoots treated with fungal extracts. We suggest that elicitation of agarwood with MeJA is a better choice for agarwood induction in *A. malaccensis* shoot cultures, as it resulted in the accumulation of some important chemical constituents.

## Declarations

### Author contribution statement

Ahmad Faizal, Rizkita Rachmi Esyanti: Conceived and designed the experiments; Analyzed and interpreted the data; Contributed reagents, materials, analysis tools or data; Wrote the paper.

Nisaa Adn'ain, Silmi Rahmani, Alda Wydia Prihartini Azar: Performed the experiments; Analyzed and interpreted the data; Wrote the paper.

Iriawati: Analyzed and interpreted the data; Contributed reagents, materials, analysis tools or data; Wrote the paper.

Maman Turjaman: Analyzed and interpreted the data; Wrote the paper.

### Funding statement

This work was supported by ITB research grant through P3MI program 2018.

### Data availability statement

Data included in article/supplementary material/referenced in article.

### Declaration of interests statement

The authors declare no conflict of interest.

### Additional information

No additional information is available for this paper.

## Appendix A Supplementary data

E-Appendices-2021-02-14_V2 E-Appendices-2021-02-14_V2
